# Ketogenic diet treatment for super-refractory status epilepticus in the intensive care unit: feasibility, safety and effectiveness

**DOI:** 10.3389/fneur.2024.1517850

**Published:** 2025-01-13

**Authors:** Yishu Ren, Mengyao Zhang, Xinxiao Fu, Yan Zhang, Fang Liu, Chenglin Wu, Haiyan Shi, Fei Tian, Gang Liu, Yicong Lin, Yingying Su, Weibi Chen

**Affiliations:** ^1^Department of Neurology, Xuanwu Hospital Capital Medical University, Beijing, China; ^2^Department of Neurology, Qingyuan Hospital of Traditional Chinese Medicine Affiliated to Guangzhou University of Traditional Chinese Medicine, Qingyuan, China; ^3^Department of Neurology, Beijing Fengtai You’anmen Hospital, Beijing, China

**Keywords:** ketogenic diet, status epilepticus, refractory status epilepticus, encephalitis, intensive care unit

## Abstract

**Background and aims:**

To investigate the feasibility, safety and effectiveness of the ketogenic diet (KD) for super-refractory status epilepticus (SRSE) in the intensive care unit (ICU).

**Methods:**

We conducted a prospective investigation on patients with SRSE treated with the KD. The primary outcome measures were ketosis development as a biomarker of feasibility and resolution of SRSE as effectiveness. KD-related side effects were also investigated.

**Results:**

Twelve patients (9 females and 3 males) with SRSE, with a median age of 34 years [range 16–69, interquartile range (IQR) 18–52], were treated with a KD. The median duration of SRSE prior to KD treatment was 21 days (range 4–46). SRSE resolved in 75% (9/12) of patients at a median of 3 days (range 1–18) after KD initiation. Among the nine KD responders, all were successfully weaned off anesthetic agents at a median of 16 days (range 4–32) after KD initiation, and all were also successfully weaned off ventilator. Side effects varied, and included gastrointestinal intolerances, malnutrition and metabolic abnormalities, electrolyte disturbance, and acute weight loss, although most of them could be corrected. No patient died due to KD, and neurofunctions continued to improve under KD therapy.

**Conclusion:**

The KD may be feasible and effective for the treatment of SRSE in the ICU. Moreover, it is relatively safe. However, there are numerous adverse events that can be corrected under close monitoring.

## 1 Introduction

Status epilepticus (SE), defined as either continuous seizure activity or two or more sequential seizures without recovery of consciousness between seizures, is a medical emergency associated with a significant morbidity and mortality. In some patients with SE, seizures do not respond to antiseizure medications (ASMs) and require anesthetic therapy. If seizures persist 24-h post initiation of anesthesia or reoccur after anesthesia is weaned, it is considered super-refractory status epilepticus (SRSE) (1), which may result in a delayed, prolonged increase in intracranial pressure, cardiorespiratory and metabolic crisis, and a substantial mortality rate of 23–57% (2, 3).

Current available evidence for the treatment of SRSE is lacking. In the case of drug treatment failure, immunotherapy such as hormones, gamma globulin, and plasma exchange, may be given when the etiology of SRSE is presumed to be autoimmune. Alternative therapies such as hypothermia, surgical resection, ketogenic diet therapy and neuromodulation (including vagus nerve stimulation, deep brain stimulation, electroconvulsive therapy and transcranial magnetic stimulation) may also be tried, all with varying levels of success (1).

Among them, the ketogenic diet (KD) is high-fat, low-carbohydrate diets that mimic a fasting state and induce ketone body production through fat metabolism. The exact mechanism of KD is still controversial, which is the focus of current research. The antiepileptic effect of KD may be related to a variety of mechanisms, including neurotransmitters, ion channels, g protein-coupled receptors, gut microbiota, mitochondria, DNA methylation, inflammation, and so on (4–10). Studies have shown that, ketone bodies may act by activating various synergistic pathways, which are still being evaluated and chronic ketosis is thought to stabilize and reduce synaptic hyperexcitability in order to conserve energy (11). Moreover, The KD has been shown to exhibit antiepileptogenic properties (12, 13) and has become a well-established treatment for pediatric drug-resistant epilepsy. Recently, emerging evidences have indicated that the KD may also be beneficial for SRSE (14–21), not only for children but also for adults. However, most evidence has come from case reports, case series, or retrospective studies (14, 16, 18, 19). Currently, there are limited prospectively collected data on practical considerations and long-term outcomes of KD in patients with SRSE in the intensive care units (ICUs), such as a study of ketogenic diet treatment of SRSE in the pediatric intensive care unit confined to children (21). Moreover, some studies relied on only urinary ketosis (17–19), which does not correlate well with seizure control (22–24). In ICUs, coexistent medical problems specific to critically ill patients may act as obstacles during KD therapy. Such challenges include decreased gastrointestinal (GI) function due to a low level of consciousness and immobility; prolonged coma therapy; increased susceptibility to infection; malnutrition and metabolic disorders; and complex interactions between the KD and concomitant treatments. Therefore, many issues remain to be addressed for the management of these SRSE patients in the critical care setting, and more data are required to assess the practice of the KD therapy in adults with SRSE patients in ICUs. Here, we carried out a single-center prospective study of the administration of the KD to patients who had SRSE in the ICU, and then presented our findings about the efficacy and adverse effects of the KD. Simultaneously, short-term (during hospitalization) tolerability and long-term (3 months after KD) tolerability are also evaluated.

## 2 Methods

### 2.1 Design and patients

This is a prospective study executed in the neurocritical care unit of Xuanwu Hospital, Capital Medical University (Brain Injury Evaluation Quality Control Centre of National Health and Family Planning Commission), that mainly evaluates whether adult patients with SRSE in the ICU can have the development of ketosis and resolution of SRSE using a standardized KD approach. The inclusion criteria were as follows: (1) age between 14 and 80 years; and (2) patients diagnosed with SRSE when the conventional treatment of SE (ASMs including intravenous benzodiazepines, valproate, levetiracetam, or phenobarbital) and coma therapy (anesthetic agents including midazolam or propofol, for more than 24 h) all proved to be unsuccessful. The exclusion criteria were as follows: (1) fatty acid transport or oxidation disorder, pyruvate carboxylase deficiency, and porphyria; (2) inability to tolerate enteral feeding, including ileus; (3) propofol infusion within 24 h; (4) hemodynamic or cardiopulmonary instability (systolic blood pressure <90 mmHg, diastolic blood pressure <60 mmHg); (5) liver failure [aspartate aminotransferase (AST), alanine aminotransferase (ALT), ammonia >5× upper limits of normal, total bilirubin >15 mg/dL, bilirubin >5 mg/dL]; (6) pancreatitis; (7) pregnancy; and (8) unstable metabolic condition (sodium >150 moL/L, glucose <3.1 mmoL/L, pH <7.2). Prior to recruitment for the KD initiation, all patients failed to respond to at least three ASMs and one anesthetic agent. Signed informed consent was given from their relatives, and all patients underwent continuous EEG monitoring during KD therapy. This study was approved by the Ethics Committee of Xuanwu Hospital, Capital Medical University.

### 2.2 Diet initiation and monitoring

The KD protocol is available in Appendix 1. After screening for contraindications, 4:1 ketogenic liquid formulas (Shenzhen Zeneca Biological Technology Co., Ltd.) were used to initiate the diet continuously at 33% of the goal via naso-enteric tube and were then increased to the goal (25–30 kcal per kg body weight per day) within 72 h. Blood glucose was monitored with fingersticks every 4 h or as needed based on the clinical course. Serum beta-hydroxybutyrate and urine ketones were recorded every 12 h. We also recorded diet information, as well as adverse events and benefits noted with the KD. The feeding tube was extubated and the diet was advanced to bolus feeding when the patient’s status epilepticus was terminated, the withdrawal of anesthetics was completed, the patient’s awakening Glasgow Coma Scale (GCS) was greater than 12, the water swallow test was grade 2 or lower, and the patient was able to perform mouth opening and swallowing.

After discharge, patients were followed up every month. Clinical visits consisted of clinical evaluation, weight assessment, and discussion of diet efficacy and side effects with caregivers. Serum beta-hydroxybutyrate and blood glucose every other day and laboratory investigation every month were collected at follow-up visits.

### 2.3 Data collection

The baseline data collected included demographics and clinical information such as medical history, SE types and durations, etiology of SE and its complications prior to the KD, and concomitant use of ASMs.

The primary outcome included the development of ketosis and resolution of SE. To assess the feasibility of KD in patients in the ICU, changes in the levels of serum glucose, beta-hydroxybutyrate, and urine ketones were monitored. To estimate the effect and tolerance of KD, days to resolution of SRSE, clinical evaluation (Glasgow Coma Scale, GCS; modified Rankin Scale, mRS; and Full Outline of Unresponsiveness, FOUR) scores, relevant laboratory data (arterial blood gas, complete blood count, liver and renal function tests, lipid panel, lipase, amylase, ammonia, prealbumin, blood electrolyte and trace elements, cardiac enzyme level, etc.), imaging findings, and continuous electroencephalography (EEG) recording alone with EEG data at discharge and during follow-up were all collected. Other data about the ability to wean off anesthetics and intravenous ASMs after the KD initiation, and outcomes at discharge and 3 months after the KD were also documented.

### 2.4 Statistical analysis

Statistical analyses were performed with the statistical software SPSS 22.0 (IBM Corporation, Armonk, NY, United States). Patient characteristics were systematically summarized by presenting categorical variables as frequencies with their respective percentages and continuous variables as medians, accompanied by their respective ranges and interquartile ranges (IQRs).

## 3 Results

From July 2020 to April 2024, we prospectively recruited 12 patients (9 females and 3 males) with SRSE and implemented ketogenic treatment. The demographic and baseline characteristics are described in Table 1. All patients were diagnosed with SRSE before KD initiation. Eight patients were diagnosed with cryptogenic new-onset refractory status epilepticus, two with virus encephalitis, two with encephalopathy. The median SE onset age was 34 (range 16–69, interquartile range [IQR] 18–52) years, with a median admission GCS score of 5 and a median FOUR score of 5. Prior to KD therapy, all patients were in a comatose state and 11 of them were assisted with mechanical ventilation. The median duration of SE prior to the KD was 21 days (range 4–46).

**Table 1:** Demographics and characteristics of ketogenic diet patients with SRSE on admission.

Patient ID	Patient 1	Patient 2	Patient 3	Patient 4	Patient 5	Patient 6	Patient 7	Patient 8	Patient 9	Patient 10	Patient 11	Patient 12
Age/sex	16/M	59/F	25/F	37/F	17/M	56/F	18/F	17/M	33/F	69/F	50/F	34/F
SE etiology	NORSE^a^	Hypoglycemic encephalopathy	NORSE^a^	Encephalitis (HSV-1)	NORSE^a^	NORSE^a^	NORSE^a^	NORSE^a^	NORSE^a^	NORSE^a^	Encephalomyopathy	Encephalitis (enterovirus)
Types of SE	NCSE	NCSE	GCSE + NCSE	GCSE	GCSE	GCSE	GCSE	GCSE	GCSE	GCSE	GCSE + EPC	GCSE + NCSE
Pre-KD SE duration, d	25	4	12	5	22	11	43	29	43	46	20	17
Brain MRI/CT	Abnormal signals in the diffuse cortex	Multiple abnormal signals in the brain (basal ganglia, cortex)	Abnormal signals in both insulae and temporal lobes	Abnormal signals in the right insula	Normal	Abnormal signals in the diffuse cortex	Abnormal signals in the diffuse cortex	Abnormal signals in both insulae and temporal lobes	Swelling of brain tissue (CT)	Symmetrical flair hyperintensity in both brachium pontine and cerebellar hemispheres	Abnormal signals in bilateral frontal temporal lobe, right insula and the left parietal lobe gyrus	Abnormal signals in the external capsule/hippocampus bilaterally
Interictal EEG	Multifocal origin discharge	Discharge prominent in the right hemisphere	Multifocal origin discharge	Discharge prominent in the right hemisphere	Multifocal origin discharge	Generalized discharge	Generalized discharge	Discharge prominent in the right hemisphere	Generalized discharge	Discharge prominent in the left hemisphere	Discharge prominent in the right hemisphere	Multifocal origin discharge
Maintenance KD ratio/route of diet	2:1/NG + NJ enteral	3:1/NG enteral	2.6:1/NG + NJ enteral	2:1/NG + NJ enteral	4:1/NG enteral	2.6:1/NG + NJ enteral + parental	2.6:1/NG + NJ enteral	2:1/NG + NJ enteral	4:1 NG + NJ	3:1 NG + NJ	2.6:1 NG + NJ	2:1 NG + NJ
Pre-KD MV/intubated (Y/N)	Y/Y	N/Y	Y/Y	Y/Y	Y/Y	Y/Y	Y/Y	Y/Y	Y/Y	Y/Y	Y/Y	Y/Y
Pre-KD ASMs (IV)	DZP, VPA, PHB	DZP, VPA, LEV	DZP, VPA, LEV, PHB	DZP, VPA, PHB	DZP, VPA, LEV, PHB	DZP, VPA, LEV, PHB	DZP, LEV, PHB	DZP, LEV, PHB	DZP, PHB	VPA, LEV, PHB,	DZP, PHB	DZP, VPA, LEV, PHB
Pre-KD ASMs (oral)	LEV, PHB, CLO, OXZ, TPM	VPA, LEV	LEV, PHB	VPA, LZV	LEV	VPA, LEV, OXZ	LEV, PHB, CLO, perampanel	LEV, PHB, CLO, OXZ perampanel	PHB, CLO	VPA, LEV, PHB, CLO	LEV, CLO, OXZ	VPA, LEV, PHB, CLO
Pre-KD Anesthetic agents	Midazolam, propofol	Midazolam	Midazolam, propofol	Midazolam	Midazolam, propofol	Midazolam, propofol	Midazolam, propofol	Midazolam, propofol	Midazolam, propofol	Midazolam, propofol	Midazolam	Midazolam, propofol
Admit GCS score	4	7	6	6	6	3	3	6	6	3	6	6
Admit FOUR score	4	12	5	10	8	4	4	4	4	4	5	7
Pre-KD other therapy	IVIG, MP	IVIG	IVIG, MP	IVIG, MP	IVIG, MP	IVIG, MP	IVIG, MP	IVIG, MP	IVIG, MP	IVIG, MP	IVIG	IVIG, MP

KD, ketogenic diet; SRSE, super refractory status epilepticus; F, female; M, male; SE, status epilepticus; NORSE, new-onset refractory status epilepticus; NCSE, nonconvulsive status epilepticus; GCSE, generalized convulsive status epilepticus; MRI, magnetic resonance imaging; EEG, electroencephalogram; ASMs, antiseizure medications; GCS, Glasgow Coma Scale; FOUR, Full Outline of Unresponsiveness; NG; nasogastric tube; NJ, nasojejunal tube; IVIG, intravenous immunoglobulin; MP, methylprednisolone; DZP, diazepam; LEV, levetiracetam; VPA, valproic acid; PHB, phenobarbital; OXC, oxcarbazepine; TPM, topiramate.

a Denotes with no identified cause.

### 3.1 Feasibility, effectiveness, and safety of KD in patients (especially adult patients) with SRSE

**Figure 1:**
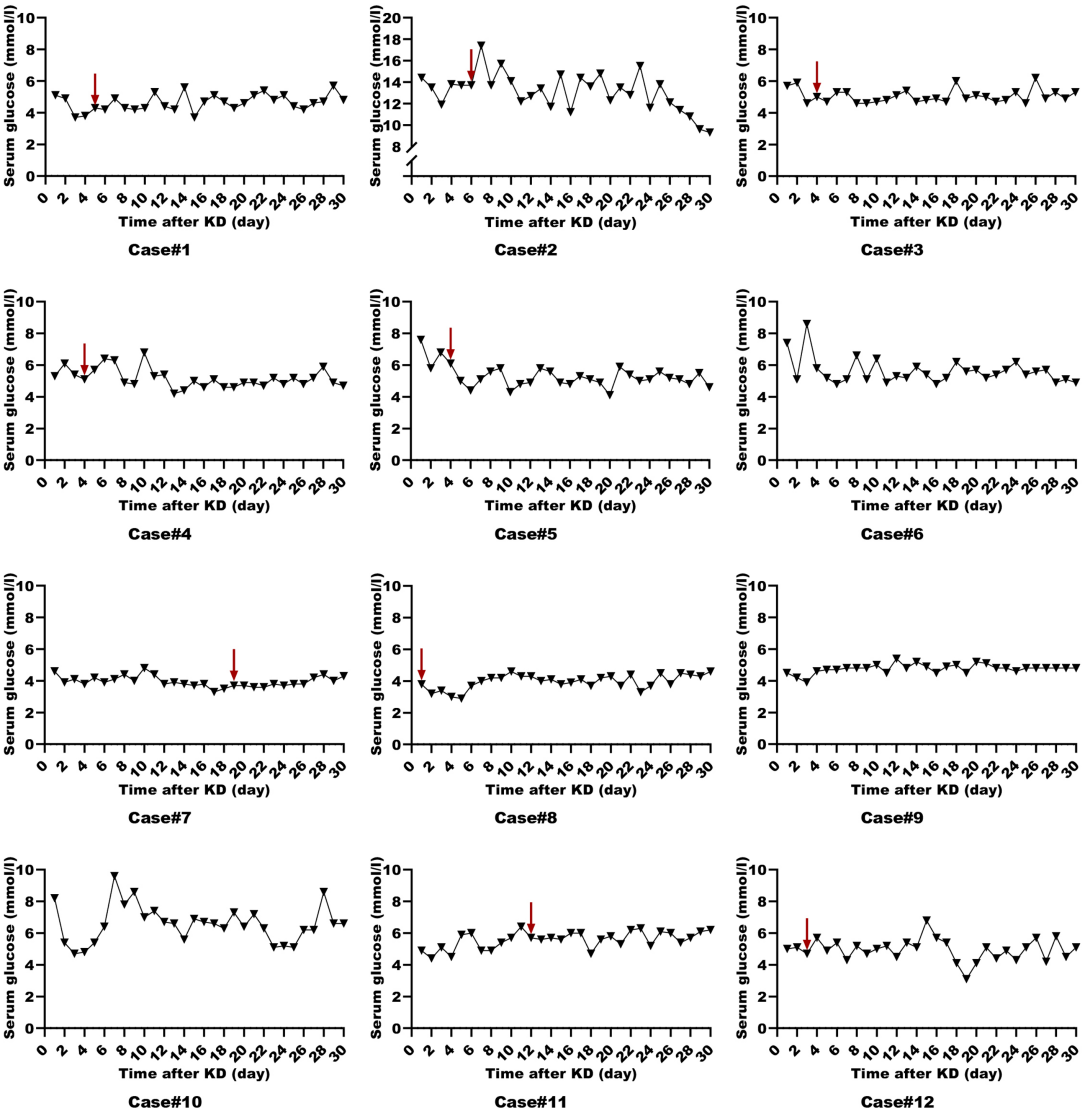
Serum glucose levels in ketogenic diet patients (First month). Serum glucose levels in 12 ketogenic diet patients during the initial month, with the red arrow indicating status epilepticus resolution.

**Figure 2:**
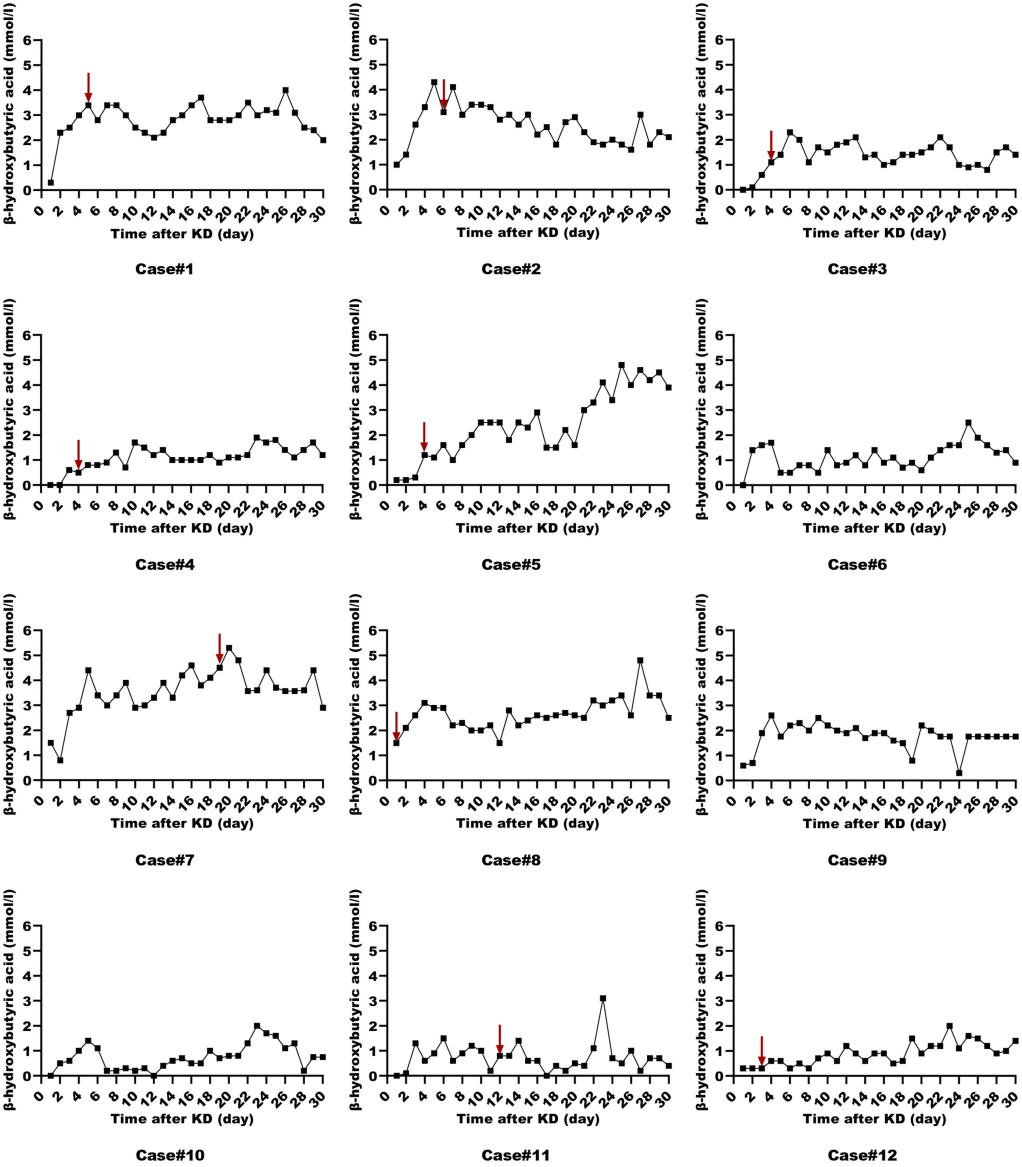
Serum beta-hydroxybutyrate levels in ketogenic diet patients (First month). Serum beta-hydroxybutyrate levels in 12 ketogenic diet patients during the initial month, with the red arrow indicating status epilepticus resolution.

**Figure 3:**
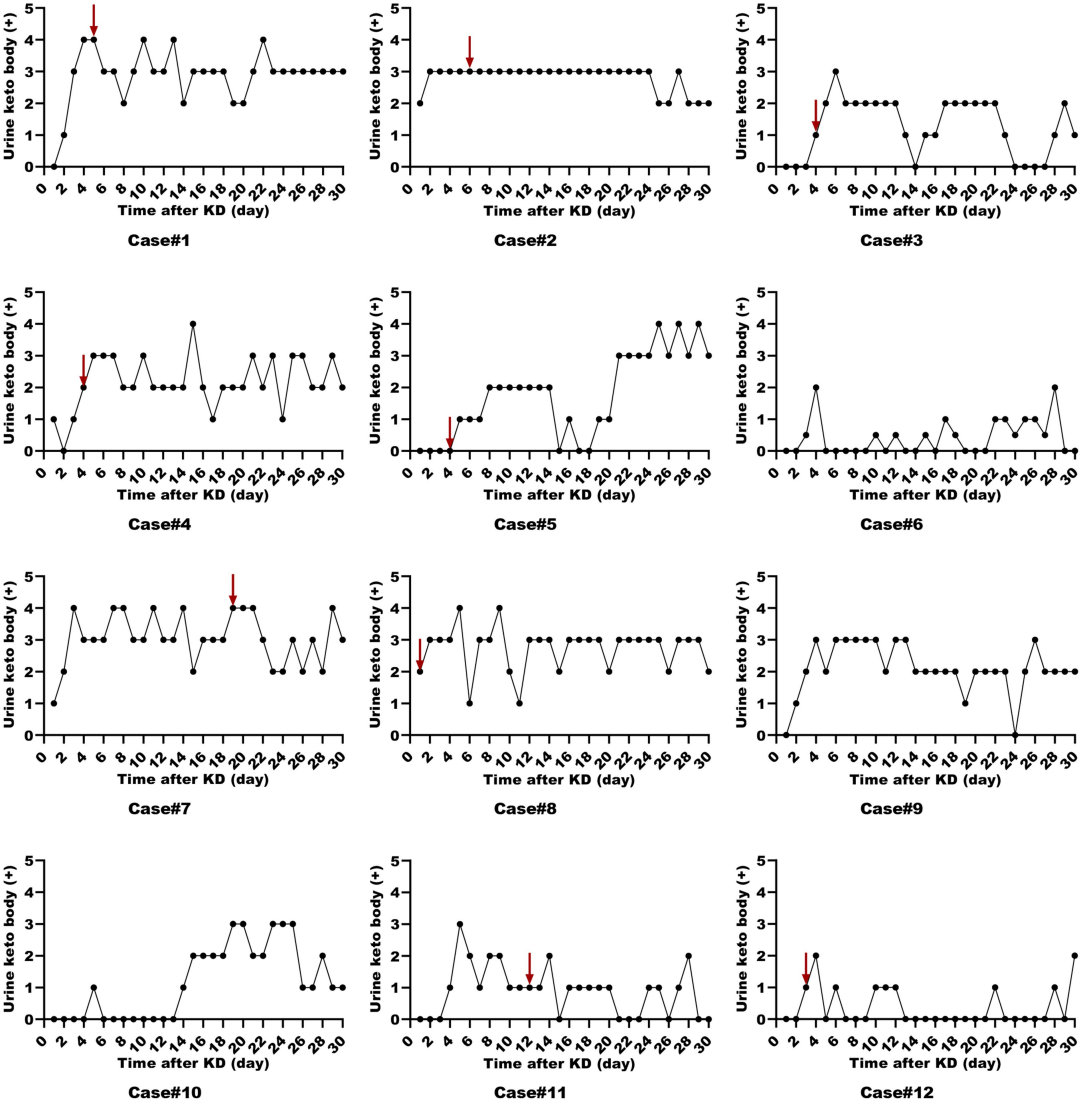
Urine ketone levels in ketogenic diet patients (First month). Urine ketone levels in 12 ketogenic diet patients during the initial month, with the red arrow indicating status epilepticus resolution.

**Figure 4:**
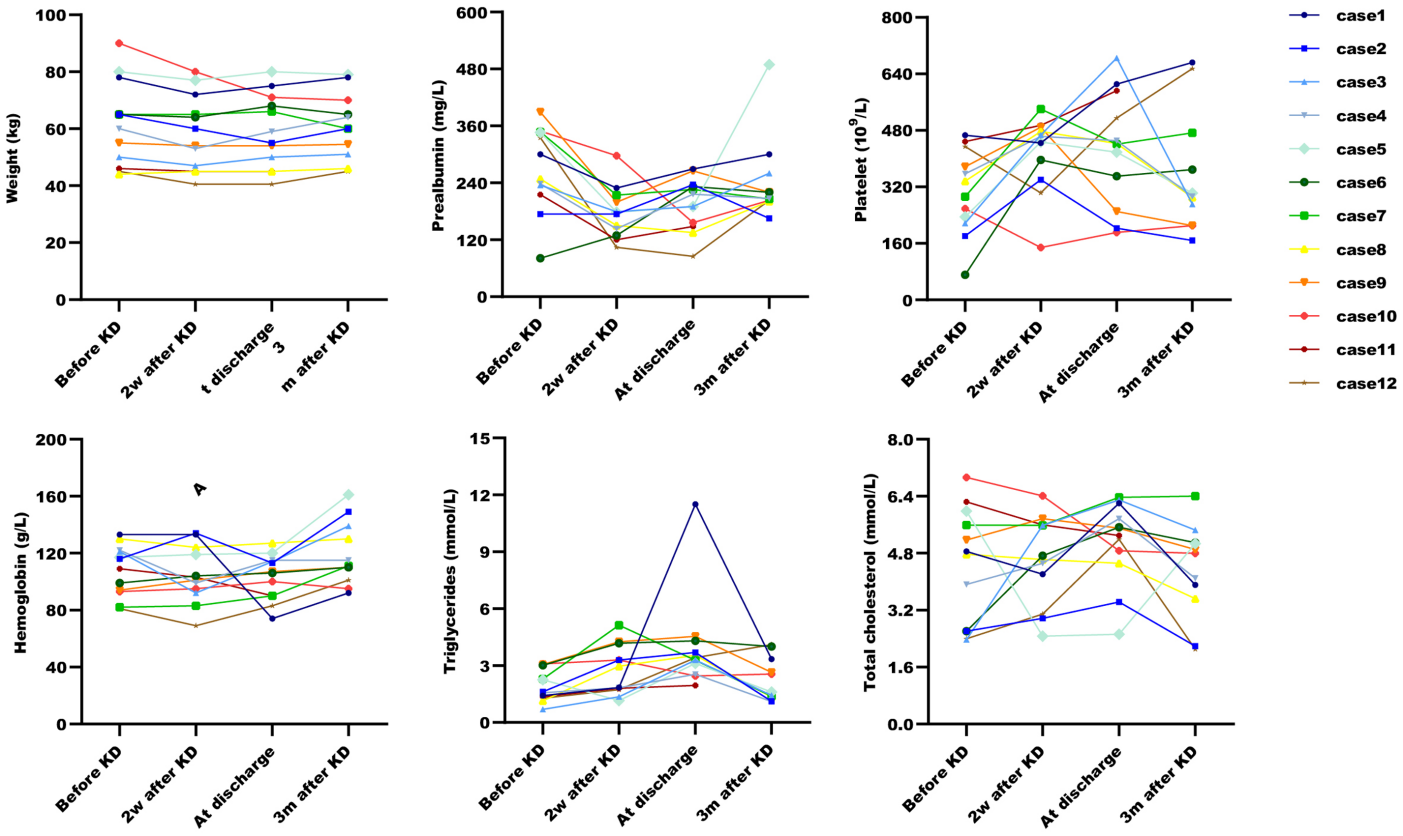
Post-ketogenic diet nutritional metabolic changes in ketogenic diet patients. Nutritional metabolic changes post-ketogenic diet across six parameters. Data for Case 11 at three months is missing due to patient’s death.

All patients were started on an enteral KD with 4:1 ratio ketogenic liquid formulas. Patient 6 was once treated with parenteral medium/long chain triglycerides to relieve severe diarrheal. Due to malnutrition, the KD ratios of those patients (except for patients 5 and 9) were finally adapted lower (range 2:1–3:1). The changes in the levels of serum glucose, beta-hydroxybutyrate, and urine ketones within the first month of KD therapy were shown in Figures 1–3. After the return of consciousness and extubation of the feeding tube, the diets for seven (patients 2, 3, 4, 5, 7, 8, and 11) of the nine KD responders (except for patients 6, 9, and 10) were able to be advanced to bolus feedings and then oral medium chain triglyceride (MCT)-KD diets. The median interval from nasogastric feeding to oral intake of KD diets was 33 days (range 9–50, IQR 27–37), while patient 1 and patient 12 stopped the ketogenic diet early due to financial problems and severe infections, respectively. Nutritional metabolic changes in body weight, levels of prealbumin, hemoglobin, platelets, triglycerides, and total cholesterol before and after the ketogenic diet were shown in Figure 4.

Nine out of twelve patients had resolution of SE at a median of 3 days (range 1–18) after KD initiation as seen in Table 2. Among the nine KD responders, all were also successfully weaned off anesthetic agents within a median of 16 days (range 4–32) after KD initiation, and finally treated with only oral ASMs, except for patient 1, whose SE relapsed during the weaning off of intravenous phenobarbital (PHB) and finally had to be maintained the condition with intramuscular PHB. In those KD responders who were ventilator-dependent, all patients were successfully weaned off ventilator.

**Table 2:** Effect of ketogenic diets.

Patient ID		Patient 1	Patient 2	Patient 3	Patient 4	Patient 5	Patient 6	Patient 7	Patient 8	Patient 9	Patient 10	Patient 11	Patient 12
Time to SE resolution in KD responders (days)		4	5	3	3	3	—	18	0	—	—	11	2
Days from KD initiation to regain consciousness		12	11	7	27	4	—	27	18	—	—	38	4
Days from KD initiation to walk independently		—	39	36	54	21	—	90	62	—	—	Death	60
Post-KD drug weaning													
	Anaesthetic agents	Y	Y	Y	Y	Y	—	Y	Y	—	—	Y	Y
	IV ASMs	SE relapse during PB weaning	Y	Y	Y	Y	—	Y	Y	—	—	Y	Y
Pre-KD MV/post-KD MV weaning		Y/Y	N/−	Y/Y	Y/Y	Y/Y	Y/−	Y/Y	Y/Y	Y/−	Y/−	Y/Y	Y/Y
mRS													
	On admit	5	5	5	5	5	5	5	5	5	5	5	5
	At discharge	5	4	3	3	3	5	5	4	5	5	4	3
	3 m after KD	5	3	2	2	1	4	3	3	2	5	6	1
MMSE													
	At discharge	Unable to assess	Unable to assess	16	14	24	Unable to assess	Unable to assess	Unable to assess	Unable to assess	Unable to assess	Unable to assess	Unable to assess
	3 m after KD	Unable to assess	16	24	18	27	Unable to assess	20	19	Unable to assess	Unable to assess	Death	28
MoCA													
	At discharge	Unable to assess	Unable to assess	11	8	19	Unable to assess	Unable to assess	Unable to assess	Unable to assess	Unable to assess	Unable to assess	Unable to assess
	3 m after KD	Unable to assess	6	18	16	22	Unable to assess	17	16	Unable to assess	Unable to assess	Death	22

KD, ketogenic diet; SE, status epilepticus; mRS, modified Rankin Scale; ASMs, antiseizure medications; MV, mechanical ventilation; MoCA, Montreal Cognitive Assessment.

In the present study, all subjects, with the exception of patient 11 (who succumbed to sepsis in an external medical facility), were administered a ketogenic diet for a minimum of 3 months. The duration of the diet was extended for those subjects experiencing increased seizure frequency during the ketogenesis phase. The ketogenic therapy duration for Patients 2, 3, 4, 5, 7, 8, and 11 was recorded as 3, 8, 6, 3, 5, 3, and 2 months, respectively. Adverse events related to the KD are listed in Appendix 2. Gastrointestinal intolerances, including constipation (1), gastric retention (1), and diarrhea (5), were seen in six patients. Additionally, malnutrition including hypoproteinemia (7) and anemia (6); as well as metabolic abnormalities including hyperammonemia (7), hyperlipidemia (8), metabolic acidosis (3), and hyperuricemia (1), commonly occur during KD. Transient electrolyte disturbance was also commonly found: eight with hypocalcaemia, four with hypophosphatemia, and five with hyponatremia. Four patients had elevated pancreatic enzymes which could be relieved with enzyme supplementation, and one patient had liver function abnormalities. Acute weight loss was a common problem in more than half of cases (patients 1, 2, 3, 4, 5, 10, and 12) initially but all could be resolved with calorie adjustments and the addition of medium-chain triglyceride ketogenic diet (MCT-KD). In the acute period, 10 patients suffered from thrombocytosis (with no thrombotic events), and most of these changes improved at a later stage. One developed urinary tract infections, another one developed sever pulmonary infection. Kidney stones were found in only one patient 2 months after KD initiation, which may have been related to the discontinuation of citrate on his own after discharge. Other reported side effects, such as episodes of hypoglycemia, did not occur in our study.

### 3.2 Functional outcomes

All patients were followed up 3 months after KD initiation. Six KD responders remained on the MCT diet at the three-month follow-up. The outcomes were variable. The patients had a median mRS score of 4 at discharge, and a median mRS score of 3 three months later. As seen in Table 2, the neurofunctions continued to improve, with the scores of Mini-Mental State Examination (MMSE) and Montreal Cognitive Assessment (MoCA) scores continuing to increase.

## 4 Discussion

Our experience supports that KD is applicable in the management of SRSE patients in the ICU. In the vast majority of critically ill SRSE patients, ketogenesis can be achieved and has a positive effect on seizure control, including resolution of SE, and weaning off of anesthetic agents, intravenous ASMs and mechanical ventilators. The KD may also cause numerous adverse effects in SRSE patients in the ICU, including gastrointestinal intolerances, malnutrition and metabolic abnormalities. However, most KD-related adverse reactions can be reversed after being carefully monitored and actively managed.

To assess whether those patients in the ICU can achieve ketosis and the level of ketosis at which SE is resolved, we utilized both urine ketones and serum beta-hydroxybutyrate as monitoring measures. Similar to previous findings, ketosis was achieved at median of 2 days (20). A study on a modified low-proportion KD in adults with SRSE reported an 83% resolution rate after a median of 9 days post-KD initiation (range 2–21) (25). However, suboptimal ketosis levels (<1.5 mmol/L) are frequently encountered (26). In our cohort, patients 6 and 10 failed to achieve adequate ketosis and consequently did not experience SE resolution following KD therapy. Intriguingly, despite initial insufficient ketosis in patients 3, 4, 5, 11, and 12, they all resolved SRSE and were successfully weaned off anesthetics, suggesting that an optimal ketosis level may not be a prerequisite for KD efficacy in these cases (25). Lowe et al. (27) demonstrated that children on a MCT-KD achieved similar seizure control despite lower urinary ketone levels. Some studies have indicated that the KD can more efficiently eliminate glutamate, the predominant excitatory neurotransmitter, and convert glutamine to γ-aminobutyric acid (GABA), the primary inhibitory neurotransmitter, thereby exerting an anti-seizure effect (28). The mechanisms by which seizures are controlled despite insufficient ketosis are not fully elucidated and may involve direct effects of ketone bodies on neuronal excitability, alterations in energy metabolism, and other potential biological mechanisms. Therefore, if ketones are low but the patient shows clinical benefit such as freedom from seizures, further action may not be required.

Previous observational studies have documented the efficacy of the KD in treating SE, with a success rate of 82% in achieving SE cessation across 31 patients (29). However, the management of SRSE in the ICU setting diverges from that of other epilepsy patients, necessitating further prospective data collection. A recent study reported a 50% response rate to KD within 7 days in eight pediatric patients with SRSE in the pediatric intensive care unit (PICU) (21). Despite this, prospective data on adult patients remain scarce. In our prospective study, significant clinical improvement was observed in nine out of 12 patients (adults and adolescents) who resolved SE following KD initiation, despite prior treatment failures. In recent studies, the authors found that resolution of SE usually occurred within 7 days (15, 30, 31). Similarly, we resolved of SE within a median of 3 days (range 1–18) after initiating KD. Notably, all nine KD-responsive patients were successfully weaned off anesthetic agents and ASMs following KD initiation. This underscores an additional potential advantage of KD in the treatment strategy for RSE: a reduction in the number of medications required for seizure management.

The safety of the KD diet in critically ill patients with RSE warrants careful consideration. KD therapy is predominantly administered via the enteral route. A meta-analysis encompassing 13 KD studies with 932 participants with drug-resistant epilepsy revealed that the most frequently reported adverse effects were vomiting, constipation, and diarrhea (32). In our cohort, gastrointestinal intolerances were observed in 58.3% of patients, manifesting as mild constipation, gastric retention and diarrhea. Given that critical RSE patients are often comatose and subjected to prolonged use of ASMs, anesthetics, and mechanical ventilation, they are predisposed to heightened gastrointestinal complications. In such scenarios, the introduction of a high-fat diet to critically ill SRSE patients may exacerbate their condition, potentially undermining the efficacy of the diet and ketosis development, as exemplified by patient 6, thus rendering the KD potentially unsafe and inefficient. For patients with impaired gastric motility, preemptive placement of a nasojejunal tube was employed to mitigate gastric retention. Moreover, administering the KD at a continuous rate minimized the risk of gastrointestinal issues. KD has been shown to affect glucose and lipid metabolism in murine models, as confirmed by Li et al. (33). Although eight patients in our study developed abnormal lipid levels, no instances of hypoglycemia were recorded, possibly attributed to the continuous feeding strategy and maintenance of adequate pancreatic and liver function during KD implementation. To optimize ketosis, we initiated treatment with the classic 4:1 KD ratio, with a temporary protein restriction of 0.5 g/kg/day in the initial phase. This approach led to hypoproteinemia in seven patients and anemia in six. Subsequent adjustments to the KD ratio with additional protein and sucrose-Fe supplementation resolved these issues. Therefore, for critical RSE patients in ICU, the initial 4:1 KD may need to be adapted later to ensure the positive nitrogen balance and meet daily nutritional requirements. Additionally, metabolic acidosis is a significant complication in ICU patients and a notable adverse effect of KD, identified as a major barrier in similar studies. Ketogenic diets increase the acid load, potentially leading to an initial phase of uncompensated metabolic acidosis and chronic low-grade metabolic acidosis (34). Untreated, this can result in dehydration and excessive weight loss, with chronic metabolic acidosis associated with complications such as nephrolithiasis and bone mineral loss (35). Consequently, metabolic acidosis must be monitored in the ICU, and the initial 4:1 KD may require adjustment in the presence of acid–base disorders to correct the imbalance caused by the diet. Interestingly, a study suggested that potassium citrate supplementation during KD therapy can prevent compensatory metabolic acidosis (36). In our study, potassium citrate was administered, and metabolic acidosis was observed in only three patients.

In our analysis of the three-month post-treatment follow-up data from all 11 KD responders, we found that although the outcomes of the patients treated with the KD were variable, the neurofunctions, including functional independent for activities of daily life evaluated with the mRS and cognitive function evaluated with the MMSE and MoCA, demonstrated continued improvement under the KD regimen, persisting even after hospital discharge. These findings align with previous reports suggesting that KD treatment may enhance both seizure control and cognitive outcomes in patients with prolonged SRSE (37).

### 4.1 Limitation

The primary limitation of our study was the small sample size, a constraint attributable to the rarity of SRSE cases within the same time frame. Our study was further limited by referral bias, as data were collected from a nationally renowned tertiary hospital specializing in neurology, which may have influenced the patient cohort. The extended period before SRSE patients were transferred to our facility also introduces potential selection bias. Additionally, the multifaceted treatment regimens for critically ill patients complicate the attribution of specific benefits to ketogenic diet (KD) intervention. Consequently, the promising effects observed must be interpreted cautiously, given the potential for confounding variables and the limited patient sample. Nonetheless, KD therapy demonstrated a rapid resolution of SRSE, a distinct advantage over other medical treatments. Future multicenter studies with larger cohorts and extended follow-up periods are essential to determine whether the observed benefits and tolerability of KD are statistically significant.

## 5 Conclusion

We conclude that the KD for SRSE may be feasible, effective, and relatively safe in the ICU. In the vast majority of critically ill SRSE patients, ketogenesis can be achieved and has a positive effect on seizure control, including resolution of SE, and weaning off of anesthetic agents and intravenous antiseizure medications (ASMs). In addition, although side effects are varied, most of them could be corrected.

## Data Availability

The original contributions presented in the study are included in the article/Supplementary material, further inquiries can be directed to the corresponding author.
