# Supplementary material for: Ketogenic diet treatment for super-refractory status epilepticus in the intensive care unit: feasibility, safety and effectiveness
Source: Front Neurol. 2025 Jan 13;15:1517850. doi: 10.3389/fneur.2024.1517850 (PMC11769800; doi:10.3389/fneur.2024.1517850)
Supplement: Supplementary file 1 [file Data_Sheet_1.docx]

Supplementary Material

# Supplementary Appendix 1. Ketogenic diet protocol for patients with SRSE in Xuanwu Hospital

| **Pre-diet** | - Fatty acid metabolism assessment - Carbohydrate intake modification: eliminate dextrose from all intravenous fluids and reformulate all medications to a low-carbohydrate variant - Assessment of gastrointestinal function and preparation for nasoenteric tube insertion - Laboratory investigations - Urological ultrasonography - Document baseline weight | - Metabolic screening: plasma amino acids, urine organic acids, free and total carnitine, and acylcarnitine profile - Laboratory investigation: arterial blood gas, complete blood count, liver and renal function tests, lipid panel, lipase, amylase, ammonia, prealbumin, urine routine, serum electrolyte and trace elements, cardiac enzyme level |
| --- | --- | --- |
| **Diet initiation** | - Initiate 4:1 ketogenic formula continuously via nasoenteric tube at 33% of goal and increase to goal (25-30 kcal/kg/day) within 72h - Add daily nutritional supplementation - Carnitine supplementation at 10-50mg/kg/day - Check serum glucose every 4 hours or as needed - Check serum beta-hydroxybutyrate and urine ketone every 12 hours | - Daily nutritional supplementation: multivitamin and minerals, potassium citrate, calcium and vitamin D - Hypoglycemia rescue: administer 12.5g intravenous dextrose for hypoglycemia (serum glucose <50 mg/dL) and recheck serum glucose levels post-infusion. |
| **Diet maintenance** | - Monitor serum glucose every 8-12 hours or as needed - Measure serum beta-hydroxybutyrate and urine ketone every 12 hours - Add MCT oil to boost ketosis - Regular laboratory surveillance (biweekly) - Anthropometric monitoring (weekly) - Post- SE EEG monitoring - Medication titration: weaning of anesthetic and sedative agents |  |
| **Follow up and discharge planning** | - Discontinue KD if there is no improvement or if intolerance occurs - Continue the KD regimen based on seizure control and tolerability before discharge. For patients on oral nutrition, initiate a modified MCT-KD with 10-20g carbohydrates/day, aiming for plasma beta-hydroxybutyrate >1mmol/l - Dietetic education and post-discharge follow-up - Monitor serum glucose and beta-hydroxybutyrate every other day - Seizure episode documentation - Weekly anthropometric recording - Monthly laboratory investigations - Weaning off the diet: if weaning diet, decrease by 0.5:1 every week |  |

KD, ketogenic diet; MCT, medium-chain triglyceride; SE, status epilepticus; EEG, electroencephalogram.
