# Supplementary material for: Ketogenic diet treatment for super-refractory status epilepticus in the intensive care unit: feasibility, safety and effectiveness
Source: Front Neurol. 2025 Jan 13;15:1517850. doi: 10.3389/fneur.2024.1517850 (PMC11769800; doi:10.3389/fneur.2024.1517850)
Supplement: Supplementary file 2 [file Data_Sheet_2.docx]

Supplementary Material

# Supplementary Appendix 2. Adverse events related to the ketogenic diet

| **Patient ID** | **Patient 1** | **Patient 2** | **Patient 3** | **Patient 4** | **Patient 5** | **Patient 6** | **Patient 7** | **Patient 8** | **Patient 9** | **Patient 10** | **Patient 11** | **Patient 12** |
| --- | --- | --- | --- | --- | --- | --- | --- | --- | --- | --- | --- | --- |
| **Adverse events** | Constipation, metabolic acidosis, hypoproteinemia, thrombocytosis, hyperammonemia, anemia, hypocalcemia, transient weight loss | Hypoproteinemia, metabolic acidosis, hyperlipidemia, hyperammonemia, anemia, urinary tract infection, hypocalcemia, hypophosphatemia, transient weight loss | Hypoproteinemia, hyperlipidemia, liver function abnormalities, hyperammonemia, anemia, thrombocytosis, coagulation abnormalities, urinary tract infections, hypocalcemia, hypophosphatemia, hyponatremia, transient weight loss | Gastric retention, diarrhea, hypoproteinemia, hyperlipidemia, hyperammonemia, elevated pancreatic enzymes (amylase), anemia, thrombocytosis, hypocalcemia, hypophosphatemia, hyponatremia, transient weight loss | Hyperammonemia, thrombocytosis, hyperuricemia, kidney stones, transient weight loss | Diarrhea, hyperlipidemia, elevated pancreatic enzymes (lipase), anemia, thrombocytosis, hypocalcemia | Diarrhea, metabolic acidosis, hyperlipidemia, thrombocytosis, elevated pancreatic enzymes (lipase), hypocalcemia, hyponatremia | Hypoproteinemia, thrombocytosis, hypomagnesemia | Diarrhea, thrombocytosis，hyperlipidemia, hyponatremia, hypokalemia | Hyperammonemia, myelosuppression(leukocyte), transient weight loss, elevated pancreatic enzymes (amylase), hypocalcemia, hyperlipidemia | Hypoproteinemia, anemia, thrombocytosis | Diarrhea, hypoproteinemia, hyperlipidemia, hyperammonemia, thrombocytosis, hypocalcemia, hyponatremia, hypophosphatemia, hypomagnesemia, transient weight loss |
